# A case of neonatal gastric perforation misdiagnosed as neonatal necrotizing enterocolitis: A literature review

**DOI:** 10.1097/MD.0000000000051231

**Published:** 2026-10-16

**Authors:** Yuyang Wang, Quan Wen, Xichun Song, Yunpeng Guo, Long Chen, Bo Chen, Gai Hang

**Affiliations:** a Department of Pediatric Surgery, Tongliao People’s Hospital, Tongliao, China; b Department of Urology, Tongliao People’s Hospital, Tongliao, China; c Department of Surgery, Inner Mongolia Medical University, Hohhot, China.

**Keywords:** gastrointestinal perforation, neonatal necrotizing enterocolitis, neonatl gastric perforation

## Abstract

**Rationale:**

Neonatal gastric perforation (NGP) is a rare but life-threatening neonatal acute abdominal condition. Its clinical and imaging manifestations may overlap with those of necrotizing enterocolitis (NEC), making accurate preoperative localization of the perforation site challenging. This case highlights the diagnostic challenges and key points in the differential diagnosis of NGP when its initial presentation resembles NEC.

**Patient concerns:**

A male infant born at 33 weeks and 5 days of gestation, with a birth weight of 2500 g, was admitted on the fourth day of life because of abdominal distension for 14 hours, feeding refusal, and cessation of flatus and stool passage.

**Diagnoses:**

Abdominal ultrasonography revealed mild bowel wall thickening, absent intestinal peristalsis, and ascites, while an upright abdominal radiograph showed a large amount of free air beneath both hemidiaphragms. Based on the infant’s prematurity, abdominal distension, feeding refusal, and imaging findings, NEC complicated by gastrointestinal perforation was initially suspected. Emergency exploratory laparotomy revealed an approximately 1-cm perforation on the posterior gastric wall near the proximal greater curvature. No intestinal ischemia, necrosis, perforation, or gastrointestinal malformation was identified. The final diagnosis was NGP.

**Interventions:**

Immediately after admission, the infant received oxygen therapy, continuous monitoring, gastrointestinal decompression, anti-infective therapy, fluid replacement, and other supportive treatment. Emergency exploratory laparotomy, repair of the gastric perforation, peritoneal lavage, and drainage were subsequently performed. Postoperatively, respiratory support, gastrointestinal decompression, total parenteral nutrition, and other supportive treatments were provided.

**Outcomes:**

The infant recovered well after surgery, gradually resumed enteral feeding, and subsequently underwent successful removal of the gastric tube and abdominal drainage tube. At the 1-month follow-up, the infant was in good general condition and weighed 2.95 kg.

**Lessons:**

NGP should be considered in the differential diagnosis of neonates presenting with rapidly progressive abdominal distension and a large pneumoperitoneum, even when ultrasonographic findings suggest NEC. Pneumoperitoneum may indicate gastrointestinal perforation but cannot independently identify the site of perforation. In neonates whose clinical presentation is not entirely consistent with NEC or whose condition progresses rapidly, early surgical evaluation and, when necessary, timely exploratory surgery may help establish the diagnosis and facilitate targeted treatment.

## 1. Introduction

Neonatal gastric perforation (NGP) is a relatively rare gastrointestinal disease that most commonly occurs in neonates aged 3 to 5 days. It is more common in premature and low-birth-weight infants and has a high mortality rate. However, the pathogenesis of NGP remains incompletely understood. The disease typically presents with abdominal symptoms as the primary initial manifestation and can rapidly progress to shock.^[1]^ Therefore, early differentiation and timely management of NGP are crucial for reducing the mortality rate associated with this disease. This study aims to analyze a case of NGP, initially diagnosed as neonatal necrotizing enterocolitis (NEC), treated at our hospital. We explored the pathogenesis, disease characteristics, and surgical intervention outcomes, providing a reference for therapeutic interventions in NGP.

## 2. Case presentation

A 4-day-old male preterm infant, born vaginally at 33 weeks and 5 days of gestation with a birth weight of 2500 g, was admitted with a 14-hour history of abdominal distension. His mother remained healthy throughout the pregnancy. The infant had no obvious cause of abdominal distension, with cessation of gas and stool passage, no nausea or vomiting, and refusal to feed. An abdominal x-ray (Fig. 1A) performed at an outside hospital suggested gastrointestinal perforation, with NEC considered. Abdominal ultrasound revealed thickening of the bowel wall, raising concerns for NEC. The patient was admitted with a diagnosis of NEC. On examination, the infant’s body temperature was 36.8°C, heart rate was 156 beats/min, respiratory rate was 28 breaths/min, and oxygen saturation was 80%. Oxygen was immediately administered via nasal cannula and face mask with continuous monitoring, followed by gastrointestinal decompression. One hour later, the oxygen saturation increased to 88%. Physical examination revealed a regular heart rhythm and clear breath sounds bilaterally. The abdomen was markedly distended, with erythema and swelling of the abdominal wall. The liver and spleen were not palpable. Abdominal percussion revealed tympany, and bowel sounds were absent. Erythema and swelling of the testes were also noted. Abdominal ultrasonography performed on admission revealed gaseous distension of several bowel loops, mild bowel wall thickening, and absent intestinal peristalsis. Fluid-filled hypoechoic areas and scattered punctate hyperechoic foci were observed within the intestinal lumen, accompanied by ascites. An upright abdominal radiograph revealed a large amount of free intraperitoneal air beneath both hemidiaphragms. The bowel loops appeared mildly rigid, with scattered intraluminal gas, but no obvious air-fluid levels or bowel dilatation were observed. Gastrointestinal perforation was suspected, with NEC considered as a possible underlying cause. Based on the infant’s prematurity, abdominal distension, feeding refusal, and ultrasonographic findings, NEC complicated by gastrointestinal perforation was initially considered on admission.

**Figure 1.**
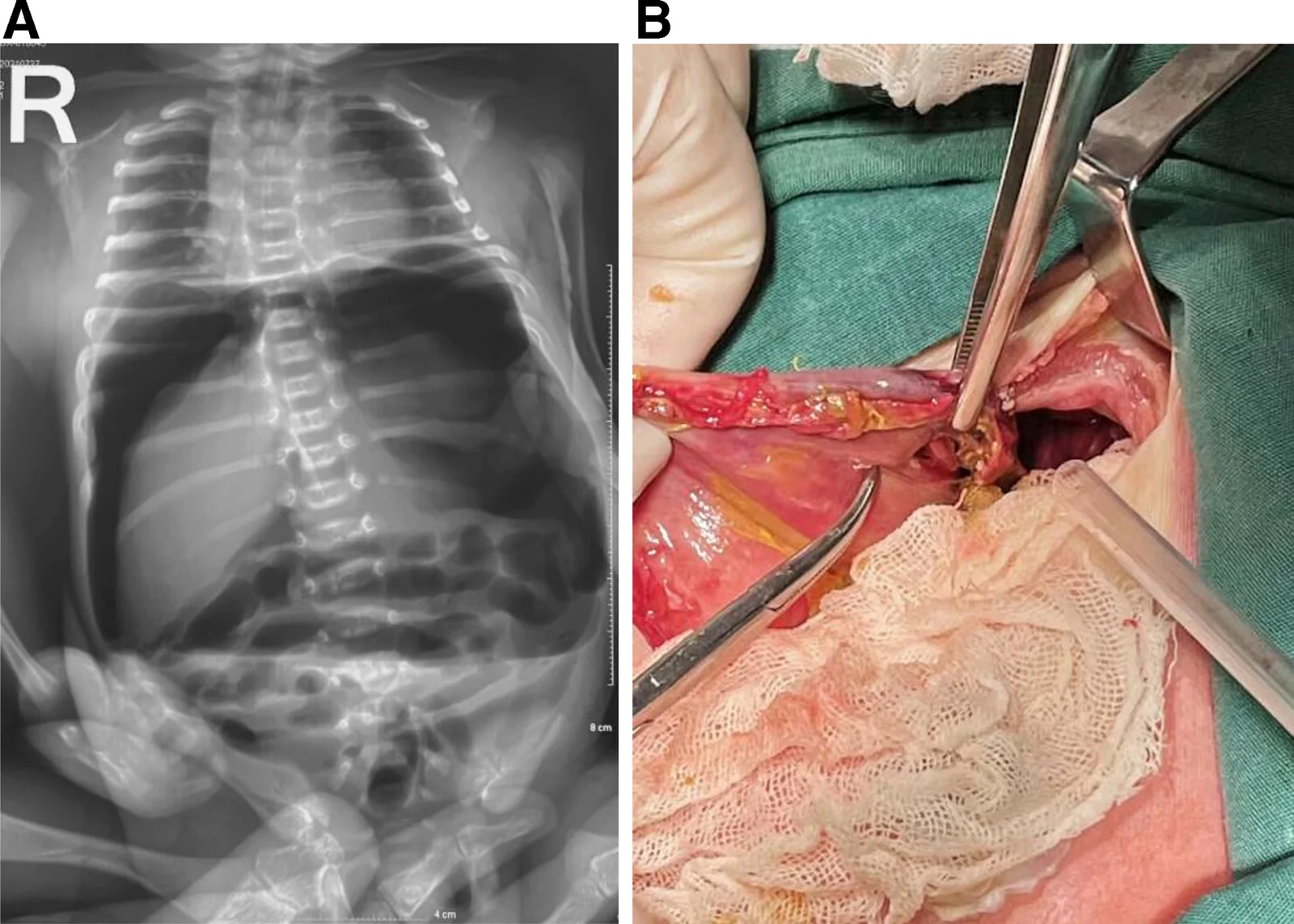
(A) Upright abdominal x-ray shows large amounts of free gas beneath the diaphragm, with scattered gas in the intestinal lumen and slightly rigid bowel morphology, as indicated by the 2 arrows. (B) The arrow indicates the site of perforation on the proximal greater curvature of the posterior gastric wall, identified during intraoperative exploration.

After admission, a gastric tube was inserted for decompression before surgery. Antibiotics and intravenous fluids were administered to control infection and correct fluid, electrolyte, and acid-base disturbances. Preoperative evaluation was completed promptly, and emergency exploratory laparotomy was performed under general anesthesia with endotracheal intubation. A 5-cm transverse supraumbilical incision was made. Upon entering the abdominal cavity, a large volume of free gas and ascitic fluid was encountered. After aspiration of the fluid, yellowish contaminated material was noted around the greater omentum. Systematic exploration of the small intestine and colon revealed no intestinal malformation, ischemia, necrosis, or perforation. The gastrocolic and gastrosplenic ligaments were opened for further exploration. An approximately 1-cm perforation with leakage of gastric contents was identified on the proximal posterior gastric wall near the greater curvature (Fig. 1B). No abnormalities of the adjacent vessels or other lesions were observed. The perforation was closed primarily with longitudinal interrupted sutures. After confirming that the repair was watertight, the abdominal cavity was repeatedly irrigated with warm normal saline. A drain was placed in the left upper abdomen adjacent to the posterior gastric wall. The gastric tube was confirmed to be appropriately positioned within the stomach, and the abdomen was closed. Postoperatively, the infant was transferred to the neonatal ward and received mechanical ventilation. As respiratory function improved, respiratory support was transitioned to nasal continuous positive airway pressure (NCPAP), followed by oxygen therapy in an incubator. Gastrointestinal decompression and anti-inflammatory therapy were continued, together with total parenteral nutrition consisting of pediatric compound amino acid injection (18AA-I; 6.20 g), fat emulsion injection (C14–24; 5.00 g), concentrated sodium chloride injection (10 mL; 0.60 g), potassium chloride injection (0.60 g), and water-soluble vitamins for injection (Soluvit; 0.15 vial). Minimal enteral feeding was initiated on postoperative day 10, and the feeding volume was gradually increased thereafter. The infant recovered well, with all clinical parameters returning to satisfactory levels, and was discharged on postoperative day 19. At the 1-month postoperative follow-up conducted by telephone, the infant’s family reported that his body weight had increased to approximately 2.95 kg (Fig. 2).

**Figure 2.**
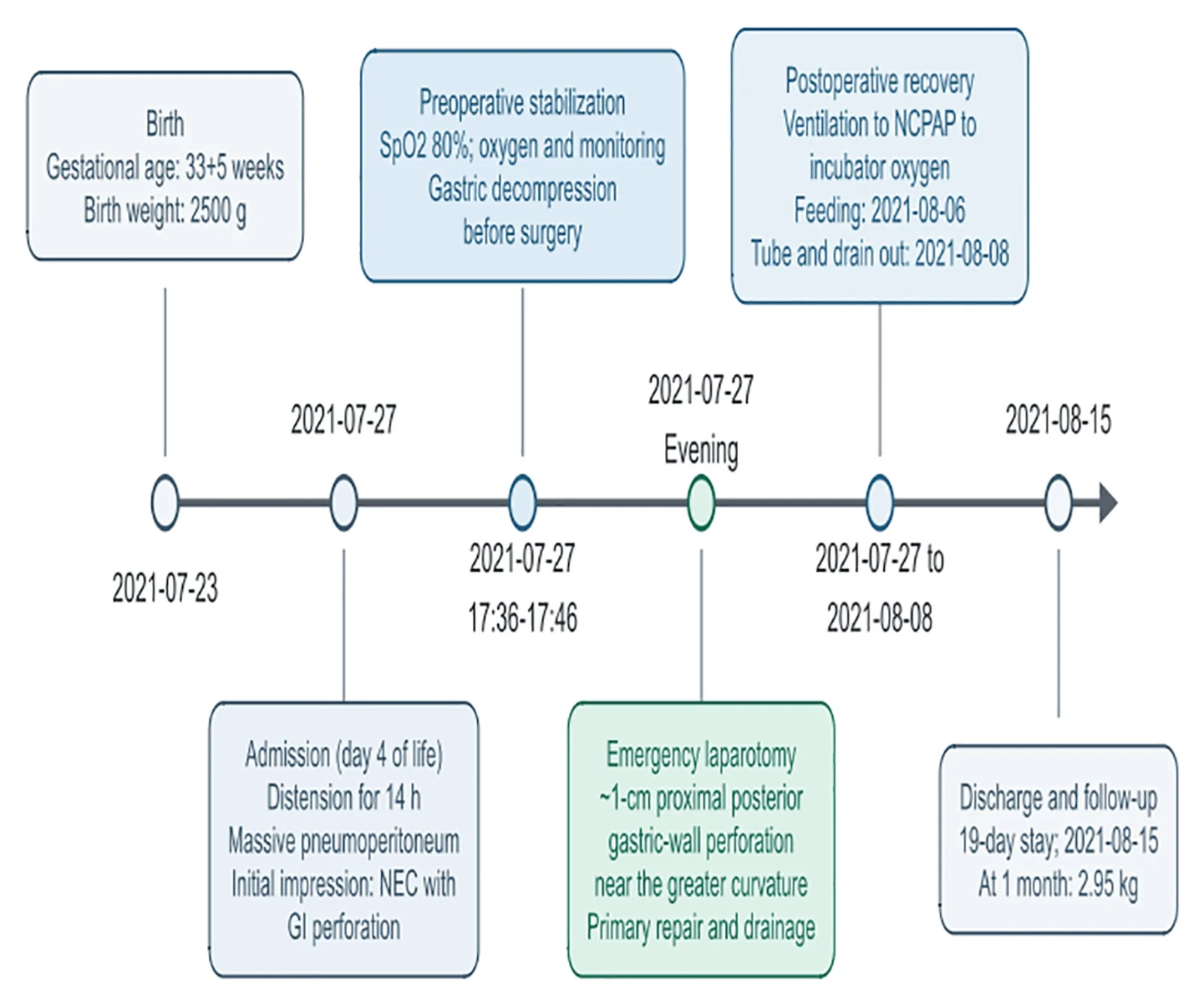
Concise clinical timeline. NCPAP = nasal continuous positive airway pressure, NEC = necrotizing enterocolitis.

## 3. Discussion

Neonatal gastric perforation (NGP) is a rare acute abdominal condition in pediatric surgery, accounting for only 7% of neonatal gastrointestinal perforations.^[2]^ However, it is life-threatening, with a mortality rate ranging from 45% to 81%.^[3]^ Huang et al.^[4]^ reported that among neonates with gastric perforation, 70.3% were premature, 61.4% had low birth weight, and only 29.7% were full-term infants. Although the incidence of NGP in full-term infants is low, the mortality rate remains high if treatment is delayed or inadequate.^[5]^ The success of treating neonatal gastric perforation depends on the timing of diagnosis and whether the infant receives timely and effective treatment. The mortality rate is closely related to the time of medical consultation. International reports indicate that the surgical survival rate within 12 hours is 45%, whereas the survival rate drops to only 25% after 12 hours.^[6]^ Therefore, timely diagnosis of neonatal gastric perforation is crucial. Only by fully understanding the imaging and clinical characteristics of gastric perforation cases can we better identify high-risk neonates in the early stages of perforation.

Since Herbut first discovered in 1941 that congenital defects in the gastric wall structure could lead to gastric perforation, numerous researchers have studied the pathogenesis of NGP.^[7]^ Most studies suggest that factors such as congenital abnormalities in gastric wall development, gastric ischemia, increased gastric pressure, improper artificial feeding, infection, stress, hypoxia, or shock leading to insufficient local perfusion contribute to NGP. Additionally, some studies have found that in neonates, maternal estrogen levels gradually decline with metabolism. Gastrin concentrations rise within the first 7 days after birth, peaking on day 7. Gastrin stimulates gastric acid secretion, leading to gastric mucosal lesions and pyloric muscle spasms, which increase gastric pressure. NGP is more common within the first 7 days, and its incidence decreases thereafter.^[8]^ However, the prevailing theory is that of developmental defects in the muscular layer of the gastric wall. This theory suggests that the muscular layer of the gastric wall extends from the lower esophagus to the gastric fundus and greater curvature in the 9th week of embryonic development. Developmental defects or vascular abnormalities during this process can lead to defects in the muscular layer. In this study, the perforation was located on the proximal posterior gastric wall near the greater curvature, and no obvious abnormalities were observed in the adjacent gastric mucosa. Although this anatomical finding may be compatible with the hypothesis of a congenital defect in the gastric muscular layer, the absence of histopathological examination prevents determination of the underlying mechanism. A study by Lin et al^[9]^ showed that the incidence of NGP is significantly higher in male than in female infants. This may be because estrogen partly inhibits gastrin levels, providing gastrointestinal mucosal protection. This suggests that sex hormone levels may be a potential high-risk factor influencing the occurrence and development of NGP, although the specific cause remains unclear and requires further research.

The early clinical symptoms of NGP are atypical and lack specificity. Affected infants may present with abdominal distension and vomiting, and some may also exhibit respiratory symptoms. Preoperative imaging to identify the location of the perforation can be challenging, leading to missed or incorrect diagnoses by clinicians.^[10]^ In the present case, the infant was born preterm and presented with abdominal distension and feeding refusal. Ultrasonography revealed bowel wall thickening, decreased intestinal peristalsis, scattered punctate hyperechoic foci, and ascites, leading to an initial suspicion of NEC. However, the upright abdominal radiograph demonstrated a large amount of free intraperitoneal air beneath both hemidiaphragms, without marked bowel dilatation or obvious air-fluid levels, suggesting gastrointestinal perforation associated with possible NEC. Nevertheless, these imaging findings could not distinguish gastric perforation from NEC-related intestinal perforation. This diagnostic difficulty is consistent with findings from previous small-sample studies, in which abdominal radiography demonstrated pneumoperitoneum in up to 84.0% of patients with NGP, while the diagnostic concordance rate of ultrasonography was reported to be as high as 89.5%.^[11]^ However, there is still a lack of effective preoperative tests that can be used to definitively diagnose NGP and reduce the occurrence of preoperative misdiagnosis and missed diagnosis. Therefore, in clinical practice, if an infant presents with the following conditions, NGP should be considered: unexplained abdominal distension and vomiting after feeding, without signs of intestinal obstruction; progressively worsening abdominal distension, rapid progression, shallow and rapid breathing, disappearance of bowel sounds, and abdominal muscle tension; premature infants with a history of respiratory distress or asphyxia after birth; full-term infants with a history of steroid use; x-ray showing pneumoperitoneum or pneumoperitoneum with liquid, especially if there is disappearance of the gastric bubble; and ultrasound showing a significant increase in pergastric fluid, with fluid also noted in the area anterior to the liver, around the gallbladder, and in the lesser omental sac.

Neonatal gastric perforation progresses rapidly, and studies have shown that infants with a time interval >24 hours from the onset of clinical symptoms to surgery tend to have a poor prognosis. This may be due to factors such as prematurity, toxin absorption, septic shock, respiratory failure, and multiple organ failure, resulting in a mortality rate of 45% to 58%.^[12,13]^ It is recommended to correct neonatal respiratory acidosis preoperatively, actively control infections, perform surgery as soon as a diagnosis is confirmed, and combine postoperative monitoring and comprehensive treatment to improve the prognosis of neonatal gastric perforation.^[14]^ NGP often occurs in the gastric fundus or greater curvature^[5]^ and may be associated with other gastrointestinal anomalies or congenital abdominal malformations, such as diaphragmatic hernia, hiatal hernia, malrotation, or Meckel’s diverticulum. Some infants may develop gastric rupture due to larger muscular layer defects and larger perforations, leading to the entry of large amounts of gas and gastric contents into the abdominal cavity, causing sudden severe abdominal distension. After confirming gastrointestinal perforation through abdominal radiography, needle decompression can be performed at the highest point between the xiphoid process and the umbilicus to relieve respiratory distress caused by abdominal distension and prevent respiratory failure. However, blind decompression should not be performed for gastric perforation not confirmed by radiography to avoid injuring the abdominal organs. The mainstream treatment for neonatal gastric perforation is the surgical removal of necrotic tissue, accompanied by reliable repair of the muscular layer of the gastric wall. After gastric perforation repair surgery, the position of the gastric tube should be confirmed within the stomach, and air should be introduced through the tube to ensure that it passes freely into the small intestine, preventing misdiagnosis of duodenal obstruction, gastrointestinal malformations, or similar diseases.^[15]^ For cases with recurrent anastomotic leaks, gastrostomy should be considered based on intraoperative findings. This effectively reduces gastric pressure. The placement of jejunal feeding tubes and early postoperative enteral nutrition can improve the survival rate of affected infants to some extent.^[16]^ Generally, feeding is initiated 5–7 days post-surgery, starting with small amounts of milk and gradually transitioning to normal breastfeeding. It is recommended to delay the start of feeding in premature infants.

## 4. Conclusion

Neonatal gastric perforation is an acute and life-threatening condition associated with a high mortality rate. Greater awareness of NGP among clinicians is essential. Early imaging evaluation to identify gastrointestinal perforation, together with prompt surgical assessment and, when indicated, timely exploration, may help avoid diagnostic and therapeutic delays. This case highlights that NGP in a preterm infant can present with clinical and ultrasonographic features overlapping those of NEC. Pneumoperitoneum may indicate gastrointestinal perforation but cannot reliably localize the perforation site. Suspected gastrointestinal perforation should prompt immediate gastrointestinal decompression and early surgical assessment. Once gastric perforation is identified intraoperatively, it should be repaired promptly, together with comprehensive perioperative management, including antibiotic therapy, respiratory support, parenteral nutrition, and close postoperative monitoring. Because histopathological examination of the gastric wall was not performed in this case, the etiology of the perforation could not be determined.

## Author contributions

**Data curation:** Yunpeng Guo, Gai Hang.

**Investigation:** Yuyang Wang, Xichun Song.

**Supervision:** Quan Wen, Bo Chen, Gai Hang.

**Writing – review & editing:** Yuyang Wang, Long Chen.
